# Prevalence and Phenotypic Correlations of GAD65 and ZnT8 Autoantibodies in Young‐Onset Diabetes: A Pilot Study in a Tertiary Centre in Bangladesh

**DOI:** 10.1002/edm2.70246

**Published:** 2026-05-21

**Authors:** Md. Saifur Rahman, A. H. M. Shadequl Islam, Mobarak Hosen, Mohammad Jahangir Alam, Nusrat Sultana, Mashfiqul Hasan, Muhammad Abul Hasanat

**Affiliations:** ^1^ Department of Endocrinology Lubana General Hospital & Cardiac Center Dhaka Bangladesh; ^2^ Department of Endocrinology Khulna Specialized Hospital Khulna Bangladesh; ^3^ Department of Endocrinology Barrister Rafique‐ul Huq Medical College Dhaka Bangladesh; ^4^ Department of Endocrinology Mugda Medical College Dhaka Bangladesh; ^5^ Department of Endocrinology Bangladesh Medical University Dhaka Bangladesh

**Keywords:** Bangladesh, GAD65 antibody, islet cell autoimmunity, young‐onset diabetes, ZnT8 antibody

## Abstract

**Introduction:**

The increasing prevalence of young‐onset diabetes in Bangladesh presents a diagnostic challenge due to overlapping clinical phenotypes. Data regarding glutamic acid decarboxylase (GAD65) and, especially, Zinc Transporter 8 (ZnT8) antibodies in the Bangladeshi population remain scarce.

**Objective:**

To determine the frequency of GAD65 and ZnT8 autoantibodies in young Bangladeshi individuals with diabetes (aged < 30 years) compared with normal glucose tolerance (NGT) controls and to evaluate the correlation between antibody titres and phenotypic variables.

**Materials and Methods:**

This case–control study enrolled 46 young individuals with diabetes (age 24 ± 4 years, BMI 23.4 ± 5.8 kg/m^2^; mean ± SD) and 42 healthy controls with NGT (age 22 ± 2 years, BMI 21.4 ± 3.3 kg/m^2^; mean ± SD) at a tertiary care hospital in Dhaka. Clinical and biochemical data were recorded, and serum GAD65 and ZnT8 antibodies were measured by enzyme‐linked immunosorbent assay (ELISA).

**Results:**

GAD65 antibody was positive in 21.7% (10/46) of the DM group compared to 11.9% (5/42) of the NGT group (*p* = 0.265). ZnT8 antibody positivity was 4.3% (2/46) in the DM group and absent in controls (*p* = 0.495). All ZnT8 antibody‐positive individuals were also positive for GAD65 antibody. Within the DM group, no statistically significant differences were detected in phenotypic variables (presenting symptoms, age, family history of DM, BMI, waist circumference, blood pressure, and blood glucose) between participants who were seropositive for any islet autoantibody and those who were seronegative for both (*p* = ns for all). A positive correlation was observed between antibody titres and HbA1c% in the DM group (*r* = 0.404, *p* = 0.005 for GAD65; *r* = 0.517, *p* < 0.001 for ZnT8).

**Conclusion:**

In this pilot cohort, GAD65 positivity was more frequent than ZnT8 positivity among young Bangladeshi individuals with diabetes, while GAD65 positivity was also detected in a proportion of NGT controls. No clear phenotypic differences were identified between antibody‐positive and antibody‐negative diabetes cases, although antibody titres showed positive correlations with HbA1c.

## Graphical abstract

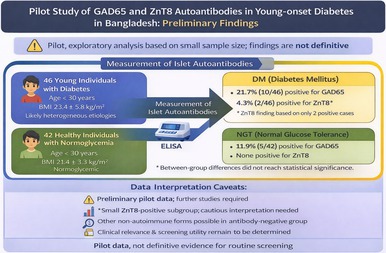

## 1 Introduction

Diabetes mellitus (DM) is one of the fastest‐growing global health crises of the 21st century, and the worldwide rate of DM in adolescents and young adults is rising rapidly [1]. In childhood and adolescence, immune‐mediated Type 1 Diabetes (T1DM) was considered the most common form; however, the increasing incidence of young‐onset Type 2 Diabetes (T2DM) has led to a complex clinical landscape [2]. Differentiating between T1DM and T2DM in young patients has become more difficult. The typical features are blending: more youth with T1DM are overweight or obese at diagnosis, while some young patients with T2DM present with lean phenotype, severe hyperglycaemia and even diabetic ketoacidosis (DKA) [3]. In fact, youth‐onset T2DM is a particularly aggressive form, marked by a rapid decline in beta‐cell function. Correct classification is vital for choosing the right treatment plan and setting long‐term management goals.

T1DM is a multifactorial autoimmune disease in which genetic predisposition and environmental triggers initiate an immune response against pancreatic beta cells. This process involves the formation of autoantibodies and the activation of autoreactive T‐cells, leading to a progressive loss of beta‐cell mass [4]. During the prodromal phase, autoantibodies targeting specific islet antigens, including insulin (IAA), glutamic acid decarboxylase (GAD65), islet antigen‐2 (IA‐2) and zinc transporter 8 (ZnT8), can be detected months or even years before the onset of overt hyperglycaemia [5]. Among these, GAD65 antibodies are present in approximately 70% of patients at diagnosis and persist for years, making them reliable markers for identifying autoimmune diabetes even years after onset [6]. Conversely, ZnT8 autoantibodies may complement established markers such as GAD65 and IA‐2 in autoimmune diabetes, but their diagnostic yield varies across populations, age groups and clinical phenotypes [7, 8, 9].

In Bangladesh, the burden of young‐onset diabetes is rising in tandem with rapid urbanization and lifestyle shifts [10]. However, clinical practice in the region often relies on phenotypic markers rather than autoimmune profiling, which is frequently limited by cost and laboratory facilities [11]. Previous studies investigating islet autoimmunity in Bangladesh have often relied on a single antibody marker, typically GAD Ab, without including a healthy control group to establish a baseline for false positivity or background autoimmunity in the normoglycaemic population [12, 13]. Furthermore, several studies continue to utilize islet cell cytoplasmic antibodies (ICA) [14, 15]. While historically significant, ICA is now considered largely obsolete in modern clinical practice due to its lack of standardization and lower sensitivity compared to specific recombinant protein‐based assays [16]. Moreover, studies have not yet evaluated ZnT8 autoantibodies in the Bangladeshi population [17], although their importance has been documented in the neighbouring countries [7, 18, 19]. It remains unclear how these autoimmune markers correlate with the biochemical profile and glycaemic severity of Bangladeshi youth. In Bangladesh and similar resource‐limited settings, young‐onset diabetes often presents with overlapping clinical features, and more definitive classification tools are not always readily available in routine care. Therefore, evaluating islet autoantibodies in a clinically heterogeneous young‐onset diabetes cohort may reflect real‐world diagnostic uncertainty and may help generate local evidence for future classification strategies. In this context, this study was designed to evaluate the frequency of GAD65 and ZnT8 autoantibodies in young patients with diabetes (< 30 years of age) compared with non‐diabetic controls at a tertiary centre in Bangladesh, under a pilot concept to assess the extent of autoimmunity. Furthermore, the study sought to assess the clinical and biochemical phenotypes of antibody‐positive individuals and evaluate the correlations between autoantibody titres and key variables.

## 2 Methods

### 2.1 Study Design and Subjects

This case–control study was conducted at the Department of Endocrinology, Bangladesh Medical University (BMU) (formerly known as Bangabandhu Sheikh Mujib Medical University), Dhaka. A total of 46 young participants (age < 30 years) with DM were enrolled in this study from January 2015 to March 2017 using non‐probability purposive sampling. In addition, 42 age‐group and sex‐matched healthy participants with normal glucose tolerance (NGT) were selected as controls. Exclusion criteria for both groups included pregnancy, endocrine disorders or the use of medications known to interfere with glucose metabolism.

### 2.2 Sample Size

The formula used to determine the sample size was: *n* = *z* ^2^ *pq*/*d* ^2^, where *z* = 1.96 (95% confidence interval), *p* = 0.08 (expected proportion based on prior regional data) [14], and *d* = 0.05 (margin of error). Although the calculated required sample size was 120, this pilot study recruited 46 cases and 42 controls, given the limitations of clinical feasibility and the pilot nature of the investigation. Therefore, the study should be interpreted as an exploratory pilot investigation, and the sample size may have limited the ability to detect moderate between‐group differences, particularly in subgroup analyses.

### 2.3 Study Procedure

Participants who fulfilled the inclusion criteria and in the absence of exclusion criteria were included in the study. Patients were recruited from the Endocrinology department of BMU, and the majority of controls were health care professionals and students (doctors, nurses, medical assistants) who voluntarily consented to participate in the study. Data were collected in a questionnaire after completion of the history and physical examination. Height, weight, waist circumference (WC) and blood pressure (BP) were measured using standard procedures. Blood glucose levels and glycated haemoglobin (HbA1c) levels were obtained from recent medical records. Then, a blood sample (5 mL) was drawn from each participant, and serum was separated and stored in the Endocrinology department at −20°C. The median storage duration before assay was approximately 6 months. Batch analysis was performed on a specific day in a single‐assay run to ensure consistency in the Department of Biochemistry and Molecular Biology at BMU.

### 2.4 Analytic Methods

Serum GAD65 and ZnT8 antibodies were measured using ELISA kits (ElisaRSR GADAb and ElisaRSR ZnT8 Ab, respectively) from RSR Ltd., Cardiff, UK. The manufacturer‐reported sensitivities and specificities for GAD65 Ab were 92% and 98%, respectively, and for ZnT8 Ab, 72% and 99%, respectively. Antibodies were considered positive if levels were ≥ 5 u/mL for GAD65 antibodies and ≥ 15 u/mL for ZnT8 antibodies.

### 2.5 Quality Assurance and Assay Workflow

Initially, 4–5 participants were enrolled for training and piloting of data and sample collection procedures. Samples were thawed only once, immediately before analysis. A quality control sample and a fixed standard were included in the assay run to assess assay precision and coefficient of variation. Laboratory personnel were blinded to case–control status.

### 2.6 Ethical Aspects

Voluntary, informed written consent was obtained from each subject after a thorough explanation of the procedure and the purpose of the study. The project was run only after approval of the Institutional Review Board of BMU (No. BSMMU/2016/1446).

### 2.7 Statistical Analysis

Data were analysed using IBM SPSS Statistics (Version 22.0; IBM Corp., 2013). Continuous variables were expressed as mean ± standard deviation (SD) or median and interquartile range (IQR), while categorical data were presented as frequencies and percentages. Group comparisons for quantitative variables were performed using the unpaired Student's *t*‐test for parametric variables and the Mann–Whitney *U* test for non‐parametric variables. The chi‐square or Fisher's exact test was used for categorical variables. Correlations between antibody titres and glycaemic markers were evaluated using Pearson correlation coefficients. *p*‐value < 0.05 was considered statistically significant.

## 3 Result

### 3.1 Baseline Characteristics

The study analysed 46 young individuals with DM and 42 with NGT. Participants in the DM group were older (23.5 ± 4.1 years vs. 21.8 ± 1.7 years, *p* = 0.009), while sex distributions were statistically similar between the two groups. Patients with DM had significantly higher WC, waist‐to‐hip ratio and diastolic BP than controls (*p* < 0.05 for all). DM group had a trend of higher BMI (23.4 ± 5.8 vs. 21.4 ± 3.3 kg/m^2^, *p* = 0.051). Family history of diabetes and acanthosis nigricans were significantly more prevalent in the DM group (Table 1).

**TABLE 1:** Characteristics of study participants (*N* = 88).

Characters	Group		*p*
	DM (*n* = 46)	NGT (*n* = 42)	
Age (mean ± SD, years)	23.5 ± 4.1	21.8 ± 1.7	**0.009**
BMI (mean ± SD, kg/m^2^)	23.4 ± 5.8	21.4 ± 3.3	0.051
Waist circumference (mean ± SD, cm)	81.1 ± 13.1	74.5 ± 8.0	**0.005**
Waist‐to‐hip ratio	0.89 ± 0.04^b^	0.83 ± 0.06	**< 0.001**
Sex			
Male	26 (56.5%)	21 (50.0%)	0.669
Female	20 (43.5%)	21 (50.0%)	
Family history of DM	30 (65.2%)	7 (16.7%)	**< 0.001**
Socio‐economic status			
High	7 (15%)	11 (26.2%)	**< 0.001**
Middle	19 (41.3%)	30 (71.4%)	
Low	20 (43.5%)	1 (2.4%)	
Acanthosis nigricans	14 (30.4%)	5 (11.9%)	**0.041**
Systolic BP (mean ± SD, mm of Hg)	115.0 ± 15.7	113.3 ± 15.3	0.616
Diastolic BP (mean ± SD, mm of Hg)	76.4 ± 9.7	69.1 ± 10.4	**0.001**
Fasting plasma glucose (mmol/L, mean ± SD)	13.0 ± 6.0	5.0 ± 0.4	**< 0.001**
2‐h plasma glucose (mmol/L, mean ± SD)	19.8 ± 7.7	6.0 ± 0.6	**< 0.001**
HbA1c (%, mean ± SD)^a^	9.7 ± 2.8	—	—

*Note:* Within parentheses are percentages over the column total. *p*‐value was measured by Student's *t*‐test and *χ* ^2^ test for quantitative and qualitative variables, respectively.

Abbreviations: BMI, body mass index; BP, blood pressure.

^a^ Measured in the DM group only.

^b^ *Note:* Values in bold indicate statistically significant *p*‐values (*p* < 0.05).

### 3.2 Clinical Presentation of Diabetes

Among the participants with DM, 45.7% had classic hyperglycaemic symptoms, 37% were asymptomatic and 10.9% had nonspecific symptoms. Three individuals with diabetes had a history of hyperglycaemic emergency. More than 50% had DM duration < 1 month, 23.9% between 1 and 12 months, and 21.7% had DM duration > 12 months (Table 2).

**TABLE 2:** Presenting symptoms of individuals with diabetes at diagnosis (*n* = 46).

Variables	Frequency (*n*)	Percentage
Presenting symptoms		
Asymptomatic	17	37.0
Hyperglycaemic symptoms	21	45.7
Diabetic emergency	3	6.5
Others	5	10.9
Duration of diabetes		
< 1 month	25	54.3
1–12 months	11	23.9
> 12 months	10	21.7

*Note:* Others = non‐classic hyperglycaemic symptoms.

### 3.3 GAD65 and ZnT8 Antibodies in DM and NGT

GAD65 antibody positivity was observed in 21.7% (10/46) of the DM group and 11.9% (5/42) of the NGT group (*p* = 0.265). On the other hand, 4.3% (2/46) of participants with diabetes tested positive for ZnT8 antibody, whereas none in the control group tested positive (*p* = 0.495) (Table 3). All participants who were positive for the ZnT8 antibody were also positive for the GAD65 antibody.

**TABLE 3:** GAD Ab and ZnT8 Ab positivity in DM and NGT groups (*N* = 88).

Findings		Group		*p*
		DM (*n* = 46)	NGT (*n* = 42)	
		*n* (%)	*n* (%)	
GAD Ab	Positive	10 (21.7%)	5 (11.9%)	0.265
	Negative	36 (78.3%)	37 (88.1%)	
GAD Ab titre (U/mL, median and IQR)		3.9 (3.1–4.8)	3.4 (2.9–4.3)	0.152
ZnT8 Ab	Positive	2 (4.3%)	0 (0%)	0.495
	Negative	44 (95.7%)	42 (100.0%)	
ZnT8 Ab titre (U/mL, median and IQR)		1.9 (0.9–4.7)	1.4 (0.3–3.5)	0.140

*Note:* Significance level was measured by *χ* ^2^, Fisher's Exact test and Mann–Whitney *U* test as applicable. GAD Ab: glutamic acid decarboxylase autoantibody; positive: ≥ 5 U/mL. Znt8 Ab: zinc transporter autoantibody; positive: ≥ 15 U/mL.

Abbreviation: IQR, interquartile range.

### 3.4 Phenotypic Comparison and Correlations

Within the DM group, no statistically significant differences were detected in phenotypic variables (presenting symptoms, age, family history of DM, BMI, WC, BP and blood glucose) between participants who were seropositive for any islet autoantibody and those who were seronegative for both (Table 4). In the DM group, both GAD65 and ZnT8 antibody titres showed a significant positive correlation with HbA1c (GAD65: *r* = 0.404, *p* = 0.005; ZnT8: *r* = 0.517, *p* < 0.001). No significant correlations were detected between antibody levels and FPG, 2 h‐PG or BMI in either group (Table 5).

**TABLE 4:** Clinical characteristics according to antibody positivity (GAD65 and/or ZnT8) of participants with DM (*N* = 46).

Characters	Group		*p*
	Ab positive (*n* = 10)	Ab negative (*n* = 36)	
Age (mean ± SD)	24.4 ± 4.4	23.3 ± 4.0	0.459
Age group			
≤ 20 years or less	2 (20.0%)	9 (25.0%)	1.000
> 20 years	8 (80.0%)	27 (75.0%)	
BMI (mean ± SD)	25.1 ± 8.6	22.9 ± 4.9	0.295
Waist circumference (mean ± SD, cm)	85.4 ± 16.9	79.9 ± 11.8	0.242
Acanthosis nigricans	3 (30.0%)	11 (30.6%)	1.000
Family history of DM	7 (70.0%)	23 (63.9%)	1.000
Duration of DM			
< 1 month	5 (50.0%)	20 (55.6%)	1.000
> 1 month	5 (50.0%)	16 (44.4%)	
Presenting symptoms			
Symptomatic	5 (50.0%)	17 (47.2%)	1.000
Asymptomatic	5 (50.0%)	19 (52.8%)	
Systolic blood pressure (mm Hg)	122.0 ± 22.5	113.1 ± 13.1	0.255
Diastolic blood pressure (mm Hg)	80.0 ± 14.1	75.2 ± 8.1	0.189
Fasting plasma glucose (mmol/L, mean ± SD)	12.3 ± 5.9	13.3 ± 6.1	0.645
2 h plasma glucose (mmol/L, mean ± SD)	17.8 ± 5.2	20.3 ± 8.2	0.371
HbA1c (%, mean ± SD)	10.6 ± 4.0	9.5 ± 2.6	0.316

*Note:* Significance level was measured by Student's *t*‐test and *χ* ^2^ test/Fisher's Exact test for quantitative and qualitative variables, respectively. Values in bold indicate statistically significant *p*‐values (*p* < 0.05).

Abbreviations: Ab, antibody; DM, diabetes mellitus; HbA1c, haemoglobin A1c; SD, standard deviation.

**TABLE 5:** Correlation of GADAb and ZnT8Ab with FPG, 2 h‐PG, HbA1c, BMI (*N* = 88).

Determinants of ‘*r*’	DM (*n* = 46)		NGT (*n* = 42)	
	*r*	*p*	*r*	*p*
GAD Ab vs. FPG	−0.062	0.683	0.011	0.947
GAD Ab vs. 2 h‐PG	0.045	0.770	0.119	0.454
GAD Ab vs. HbA1c	0.404	**0.005**	—	—
GAD Ab vs. BMI	−0.260	0.081	−0.130	0.412
ZnT8 Ab vs. FPG	0.020	0.894	0.175	0.267
ZnT8 Ab vs. 2 h‐PG	0.187	0.223	0.293	0.060
ZnT8 Ab vs. HbA1c	0.517	**< 0.001**	—	—
ZnT8 Ab vs. BMI	−0.183	0.224	0.010	0.947

*Note:* Correlation between variables was done by Pearson's correlation test.

Abbreviations: 2 h‐PG, 2‐h after 75 g glucose; BMI, body mass index; FPG, fasting plasma glucose; GAD Ab, glutamic acid decarboxylase autoantibody; WC, waist circumference; Znt8 Ab, zinc transporter autoantibody.

## 4 Discussion

In this pilot study, involving young individuals (under 30 years of age) with diabetes and NGT, the GAD65 antibody was positive in a notable portion of participants with DM (21.7%). However, a 11.9% positivity rate was also observed in healthy NGT controls. In contrast, ZnT8 antibody positivity was observed in only 4.3% of the DM group and was entirely absent in the control group. Our analysis also could not identify a significant difference in phenotypic characteristics between antibody‐positive and antibody‐negative cases. However, as the achieved sample size was smaller than the calculated target, this study had limited statistical power, particularly for subgroup comparisons. Accordingly, non‐significant findings should not be interpreted as evidence of true equivalence between groups.

The GAD65 antibody prevalence of 21.7% in our young DM group was observed to be lower than that typically reported in Western T1DM cohorts, where an antibody positivity rate of 85% in clinical T1DM, 24% in clinical T2DM, and 57% in the clinically unclassified DM were reported in 313 young Swedish patients aged 15–34 years [20]. However, the rate of GAD65 antibody positivity has been reported to range from 8% to 28% in studies conducted in Asia [12, 13, 14, 17, 21]. The variation in GAD65 antibody positivity across studies may reflect differences in recruitment strategies, case definitions, ethnicity and assay thresholds. On the other hand, ZnT8 antibody is a well‐recognized autoantigen in classic autoimmune T1DM, particularly around the time of diagnosis, and its prevalence is influenced by age at onset and disease duration. In paediatric and new‐onset T1DM cohorts, ZnT8 positivity has been reported more frequently than in other forms of diabetes [22]. In contrast, studies in adult‐onset autoimmune diabetes indicate that ZnT8 may provide complementary diagnostic information, but its prevalence is lower, and its contribution is more variable than in classical childhood T1DM [8, 9]. Furthermore, limited sensitivity has also been reported in some adult autoimmune diabetes settings, including latent autoimmune diabetes in adults (LADA), indicating that the diagnostic performance of ZnT8 is context‐dependent rather than uniform across populations [19]. In our pilot cohort, ZnT8 positivity was observed in only two participants with diabetes and in none of the controls; notably, both ZnT8‐positive individuals were also positive for GAD65. Although this pattern may suggest possible added specificity of ZnT8 in this setting, the estimate is based on very sparse events and should be interpreted with caution. Accordingly, our findings do not establish ZnT8 as a clearly more specific marker in the Bangladeshi population, and its additional diagnostic value beyond GAD65 appears limited in this cohort. Larger, better‐characterized studies are needed to determine whether ZnT8 has a meaningful adjunctive role in the classification of young‐onset diabetes in this setting. Nevertheless, the low frequency of GAD65 and ZnT8 antibody positivity observed in this study does not necessarily imply a low prevalence of autoantibodies in immune‐mediated diabetes in Bangladeshi ethnic people. Rather, these figures reflect the autoimmune burden in a heterogeneous cohort with mixed etiopathogenesis, in which T1DM, T2DM and atypical forms may coexist. The baseline characteristics of the DM group, including a mean BMI within the overweight range, a strong familial predisposition, frequent asymptomatic presentations, and a high prevalence of acanthosis nigricans and increased waist‐to‐hip ratios, collectively indicate that young‐onset diabetes in Bangladesh may not be a single entity. Instead, it represents a complex clinical spectrum.

One of the most noteworthy findings of this pilot study was the 11.9% GAD65 positivity observed in the normoglycaemic control group. This finding should be interpreted cautiously, as it suggests a background prevalence of GAD65 positivity in this Bangladeshi population, which might have important implications for local assay threshold calibration and interpretation of autoimmune markers. However, alternative explanations must also be considered. In the present study, positivity was defined using manufacturer‐recommended cut‐offs rather than a locally validated reference range, and the control group was composed largely of health care professionals and students rather than a community‐based healthy sample. In addition, serum samples were stored at −20°C and analysed in a batch of single‐assay runs; although this approach improved procedural consistency, potential pre‐analytic and batch‐related influences cannot be fully excluded. Therefore, the observed GAD65 positivity in controls should be considered a preliminary signal that requires confirmation in larger population‐based studies using locally derived cut‐offs and rigorously standardized assay procedures.

Classically, antibody‐positive patients are expected to be younger, leaner and to present with more marked hyperglycaemia. In the present study, however, we did not identify statistically significant differences in age, BMI or WC between antibody‐positive and antibody‐negative participants. This finding should be interpreted with caution, as the antibody‐positive subgroup was small, and the study may not have been sufficiently powered to detect modest phenotypic differences. A similar lack of clear distinction was reported in another study assessing GAD65 antibody in apparent T2DM patients in Dhaka [13]. Taken together, these observations suggest that, in South Asian settings where the thin‐fat phenotype and rapid urbanization have contributed to an increase in youth‐onset T2DM [23], phenotypic boundaries between autoimmune and non‐autoimmune diabetes may be less clearly demarcated than in classical descriptions. Accordingly, while our pilot data did not identify clear clinical discriminators, they also indicate that phenotypic features alone may be insufficient to confidently exclude autoimmune diabetes in young patients. It is also important to recognize that the antibody‐negative group in this cohort may have included etiologically distinct forms of diabetes, such as pancreatic and monogenic diabetes, which could not be further characterized in the absence of imaging, C‐peptide measurement and genetic evaluation.

In this study, GAD65 and ZnT8 antibody titres showed positive correlations with HbA1c levels. While this finding is of interest and reflects an increasing glycaemic burden alongside increasing antibody titres, it should be interpreted cautiously as HbA1c may be influenced by several clinical factors, including time since diagnosis, delay in initiating therapy, treatment adherence, prior insulin use and remaining β‐cell function. As these variables were not examined in an adjusted model, the present results should be regarded as observational rather than explanatory. Therefore, the association identified in our cohort does not by itself establish that higher antibody titres reflect a more severe underlying disease process. In addition, a previous study in Bangladesh did not observe a similar positive correlation, suggesting that this finding may not be consistent across cohorts [17].

One of the strengths of this study is the inclusion of ZnT8 antibody, a modern biomarker that has not been well explored in previous Bangladeshi research, and the use of a healthy control group to provide a local reference context for antibody interpretation. However, as a pilot study, it has several limitations. The sample size was small, and participants were recruited from a single tertiary care centre, which may have introduced selection bias. Furthermore, the control group was largely composed of hospital‐associated volunteers rather than the general population. We used the manufacturer's recommended cut‐off for antibodies, but future studies should establish population‐specific thresholds for the Bangladeshi population, encompassing large‐scale healthy controls. We did not measure other antibodies, such as IA‐2 or IAA. Although prior insulin exposure in many participants would have limited the interpretability of IAA, the absence of a broader antibody panel (especially IA‐2) reduced the ability to characterise autoimmune diabetes more comprehensively. More importantly, the lack of assessment of β‐cell reserve, particularly by C‐peptide, limited our ability to distinguish insulin‐deficient autoimmune diabetes from other young‐onset diabetes phenotypes. Therefore, although antibody positivity was identified in a subset of participants, the present study cannot determine the true proportion of autoimmune diabetes in this heterogeneous cohort, and antibody positivity alone should not be taken as sufficient to define a specific clinical subtype. Integrating polygenic risk scores (PRS) for T2DM or targeted genetic screening for maturity‐onset diabetes of the young (MODY) might have provided additional diagnostic clarity, although this was beyond the scope of the present pilot study. Likewise, formal assessment of insulin resistance using the Homeostasis Model Assessment (HOMA‐IR), serum ketones and pancreatic imaging could have further refined the differential diagnosis. Finally, the lack of longitudinal follow‐up prevented us from determining progression to absolute insulin requirement among antibody‐positive individuals.

Future research should transition from this pilot phase to large‐scale, multi‐centre population studies with longitudinal follow‐up to monitor β‐cell decline and insulin requirements over time. It is recommended that subsequent investigations should incorporate a broader panel of biomarkers, including C‐peptide levels and genetic screening, alongside an expanded array of autoantibodies to better characterise the polygenic heterogeneity of the Bangladeshi youth population. Future studies should determine whether standardised screening approaches for GAD65 and ZnT8 are clinically useful in selected young patients with high glycaemic burden, regardless of phenotype.

## 5 Conclusions

This pilot study identified GAD65 and/or ZnT8 autoantibody positivity in a subset of young Bangladeshi patients with diabetes and also found measurable GAD65 positivity in some normoglycaemic controls. No clear phenotypic differences were identified between antibody‐positive and antibody‐negative diabetes cases. The antibody titres showed positive correlations with HbA1c. However, this observation should be interpreted cautiously, given the small subgroup size and limited statistical power, and these associations should be regarded as preliminary and descriptive rather than mechanistic. In addition, because of the limited number of ZnT8‐positive cases, the lack of a broader autoantibody panel, and the absence of key classification measures such as C‐peptide, the study cannot determine the true prevalence of autoimmune diabetes or the diagnostic role of these markers in routine practice. Larger studies using locally validated cut‐offs and more comprehensive phenotyping are needed before firm clinical conclusions can be drawn.

## Author Contributions

**Md. Saifur Rahman:** conceptualization, writing – original draft, data curation, formal analysis, writing – review and editing. **A. H. M. Shadequl Islam:** conceptualization, data curation, writing – review and editing. **Mobarak Hosen:** conceptualization, data curation, writing – review and editing. **Mohammad Jahangir Alam:** conceptualization, data curation, writing – review and editing. **Nusrat Sultana:** conceptualization, methodology, writing – review and editing, visualization, writing – original draft. **Mashfiqul Hasan:** conceptualization, formal analysis, visualization. **Muhammad Abul Hasanat:** writing – review and editing, project administration, funding acquisition, validation, conceptualization, formal analysis.

## Funding

This research was a pilot study conducted at the Department of Endocrinology, Bangladesh Medical University (formerly known as Bangabandhu Sheikh Mujib Medical University). No specific grant from any funding agency in the public, commercial or not‐for‐profit sectors was received for this work.

## Conflicts of Interest

The authors declare no conflicts of interest.

## Data Availability

The data that support the findings of this study are available from the corresponding author upon reasonable request.
